# *VPS13D*‐related disorders presenting as a pure and complicated form of hereditary spastic paraplegia

**DOI:** 10.1002/mgg3.1108

**Published:** 2019-12-26

**Authors:** Kishin Koh, Hiroyuki Ishiura, Haruo Shimazaki, Michiko Tsutsumiuchi, Yuta Ichinose, Haitian Nan, Shun Hamada, Toshihisa Ohtsuka, Shoji Tsuji, Yoshihisa Takiyama

**Affiliations:** ^1^ Department of Neurology Graduate School of Medical Sciences University of Yamanashi Yamanashi Japan; ^2^ Department of Neurology Graduate School of Medicine The University of Tokyo Tokyo Japan; ^3^ Division of Neurology Department of Internal Medicine Jichi Medical University School of Medicine Tochigi Japan; ^4^ Department of Neurology Jichi Medical University Saitama Medical Center Omiya Japan; ^5^ Department of Neurology Toranomon Hospital Tokyo Japan; ^6^ Department of Biochemistry Graduate School of Medical Sciences University of Yamanashi Yamanashi Japan; ^7^ Department of Molecular Neurology Graduate School of Medicine University of Tokyo Tokyo Japan; ^8^ Department of Neurology International University of Health and Welfare Chiba Japan

**Keywords:** autosomal recessive hereditary spastic paraplegia, complicated form, pure form, *VPS13D*‐related disorders

## Abstract

**Background:**

Alterations of vacuolar protein sorting‐associated protein 13 (VPS13) family members including VPS13A, VPS13B, and VPS13C lead to chorea acanthocytosis, Cohen syndrome, and parkinsonism, respectively. Recently, *VPS13D* mutations were identified as a cause of *VPS13D*‐related movement disorders, which show several phenotypes including chorea, dystonia, spastic ataxia, and spastic paraplegia.

**Methods:**

We applied whole‐exome analysis for a patient with a complicated form of hereditary spastic paraplegia (HSP) and her unaffected parents. Then, we screened the candidate genes in 664 Japanese families with HSP in Japan.

**Results:**

We first found a compound heterozygote *VPS13D* mutation and a heterozygote *ABHD4* variation in a sporadic patient with spastic paraplegia. Then, we found three patients with *VPS13D* mutations in two Japanese HSP families. The three patients with homozygous mutations (p.Thr1118Met/p.Thr1118Met and p.Thr2945Ala/p.Thr2945Ala) in the *VPS13D* showed an adult onset pure form of HSP. Meanwhile, the patient with a compound heterozygous mutation (p.Ser405Arg/p.Arg3141Ter) in the *VPS13D* showed a childhood onset complicated form of HSP associated with cerebellar ataxia, cervical dystonia, cataracts, and chorioretinal dystrophy.

**Conclusion:**

In the present study, we found four patients in three Japanese families with novel *VPS13D* mutations, which may broaden the clinical and genetic findings for *VPS13D*‐related disorders.

## Graphical abstract

We identified four Japanese patients with *VPS13D*‐related disorders presenting as a pure or a complicated form of HSP.

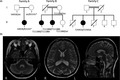

## 1 INTRODUCTION

Yeast vacuolar protein sorting‐associated protein 13 (VPS13) is the founding member of a highly conserved gene family found in all eukaryotes. In humans, there are four VPS13 orthologues, that is, VPS13A, B, C, and D (Velayos‐Baeza, Vettori, Copley, Dobson‐Stone, & Monaco, 2004). It has been reported that the mutations of *VPS13A* (OMIM *605978), *VPS13B* (OMIM *607817)*,* and *VPS13C* (OMIM *608879) lead to chorea acanthocytosis (OMIM #200150), Cohen syndrome (OMIM #216550), and parkinsonism (OMIM #616840), respectively (Kolehmainen et al., 2003; Lesage et al., 2016; Rampoldi et al., 2001; Ueno et al., 2001). Recently, *VPS13D* (OMIM *608877) was reported to be a cause of childhood onset movement disorders (Gautheir et al., 2018) and recessive ataxia with spasticity (Seong et al., 2018). According to these reports, *VPS13D* mutations lead to disorders that have a lot of symptoms including developmental delay, movement disorders, spastic ataxia, and paraparesis. Furthermore, *VPS13D* causes spinocerebellar ataxia (SCA) recessive type 4 (SCAR4, OMIM #607317) (Seong et al., 2018). The two earlier reports indicate *VPS13D*‐related disorders show various symptoms. In the present study, we report four patients in three families with *VPS13D*‐related disorders with novel *VPS13D* mutations. We present the clinical findings in three patients in two families with an adult onset pure form of hereditary spastic paraplegia (HSP), and one patient with spastic paraplegia associated with ataxia and retinochoroidal dystrophy with a childhood onset complicated form of HSP. The present study would provide an opportunity for analysis of the genotype–phenotype correlation of *VPS13D*‐related disorders.

## 2 MATERIALS AND METHODS

### 2.1 Ethical compliance

The present study was approved by the institutional review boards of Yamanashi University, Tokyo University, and Jichi Medical University School of Medicine, and written informed consent was obtained from all the participants.

**Figure 1:**
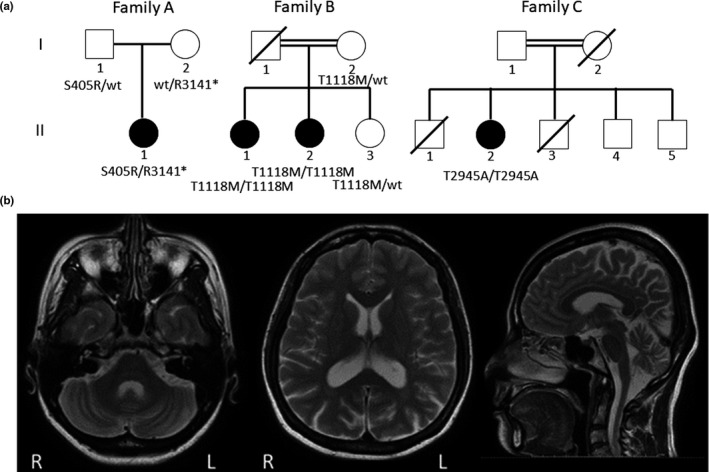
(a) Pedigree charts. Squares and circles indicate males and females, respectively. Filled symbols indicate affected individuals, whereas open symbols indicate unaffected individuals. The double lines indicate consanguineous marriage. (b) T2‐weighted brain MRI of Patient A‐II‐1. Brain MRI showed cerebellar atrophy (middle and right) and opened posterior horns of the lateral ventricles (left)

We first examined the clinical and genetic findings in Family A (Figure 1a). Patient A‐II‐1 exhibited a complicated form of HSP with cerebellar ataxia and chorioretinal dystrophy. She showed chorioretinal dystrophy at age 3, cataracts in both eyes at age 18, gait disturbance with spasticity and ataxia at age 22, and dysarthria at age 44. She underwent bilateral cataract surgery at age 26. Her score on assessment and rating of ataxia (SARA) was 19 at age 44. Furthermore, she exhibited dystonia in her neck. Brain MRI showed cerebellar atrophy and opened posterior horns of the lateral ventricles (Figure 1b). According to this clinical information, we diagnosed her as having a complicated form of HSP or spastic ataxia.

Since she was born to unaffected parents, we deduced the disease‐causing gene was due to autosomal recessive inheritance or a de novo mutation. We performed whole‐exome analysis for the patient and the unaffected parents using a Sureselect Human All Exon V5 + UTRs Kit, a Illumina Hiseq 2000 (100 bp paired end), a Burrows–Wheeler Aligner (Li & Durbin, 2009), and a GATK (Mckenna et al., 2010). We checked known genes for HSPs and SCAs by whole exome analysis for exclusive diagnosis of HSPs, Charcot–Marie–Tooth diseases, SCAs, retinal dystrophy, and cataracts. (Data S1). We also excluded known SCA genes causing repeat expansions (Data S2). We narrowed down candidate genes by means of two models of inheritance, that is, the autosomal recessive model and the de novo model, to identify the causative mutations among a lot of variations. For the autosomal recessive model, we hypothesized two patterns, a homozygous mutation or a compound heterozygous mutation. We first extracted homozygous variations in the proband and heterozygous variations in the parents for the autosomal recessive model of homozygous variations. We excluded single‐nucleotide polymorphisms by using gnomAD (Lek et al., 2016), dbSNP (Sherry et al., 2001), the Human Genetic Variation Database (Higasa et al., 2016), and 1,261 in‐house data. We narrowed down variants by filtering with a depth over 20 and variants only in exon or splicing sites. Second, we extracted two or more heterozygous variations in one gene in the proband and heterozygous variations in the parents as compound heterozygous variations. We excluded single‐nucleotide polymorphisms as if examining a homozygous mutation. Third, we extracted heterozygous variations in the proband that were not detected in the parents as de novo variations. We only picked up variants with a depth of ≧0.3 times the reference depth to avoid de novo variant errors based on next‐generation sequencing (Koh et al., 2014). We excluded single‐nucleotide polymorphisms as if examining a homozygous mutation.

We then tried to detect causative gene variations in 367 patients in the Japan Spastic Paraplegia Research Consortium and 297 collected patients with HSP in the Department of Neurology, The University of Tokyo. These patients were subjected to whole‐exome sequencing or whole‐genome sequencing.

We evaluated the functional prediction of candidate variations by means of the in silico algorithm using PolyPhen‐2 (Adzhubei et al., 2010), SIFT (Choi, Sims, Murphy, Miller, & Chan, 2012), and the PHRED‐like CADD score (Kircher et al., 2014).

After screening of candidate variations, we analyzed the clinical features of the patients. The clinical features comprised age at onset, mental development, tendon reflexes, pathological reflexes, cerebellar ataxia, dystonia, and brain MRI findings.

### 2.2 Accession numbers

C490163, LC490164, LC490165, LC490166, LC490167, LC490168, LC490169, LC490170, LC490171, LC490172, and LC490173.

## 3 RESULTS

We detected 14,475 variations in Patient A‐II‐1 on whole‐exome analysis. We did not find any known HSPs, SCAs, or cataract gene variations in Patient A‐II‐1. We found no homozygous variations and only one compound heterozygous variation in *VPS13D* under the hypothesis that the disease had occurred through the autosomal recessive mode of inheritance. Meanwhile, we found one heterozygous variation in *ABHD4* as a de novo variation. We performed direct sequencing for this family to confirm these variations. We found *VPS13D* variations (c.1215T>G, p.Ser405Arg/c.9421C>T, p.Arg3141Ter) as a compound heterozygous status and a *ABHD4* variation (c.668C>T, p.Pro223Leu) as a de novo one.

On screening of *VPS13D* and *ABHD4* variations in our data set, we found two families (Families B and C) with *VPS13D* variations (c.3353C>T, p.Thr1128Met/c.3353C>T, p.Thr1128Met, and c.8833A>G, p.Thr2945Ala/c.8833A>G, p.Thr2945Ala, respectively) (Figure 1a). We found no families with *ABHD4* variations in our data set. Variation of c.1215T>G, c.9421C>T, and c.3353C>T was not apparent on ExAC, HGVD, and iJGVD. Variation of c.8833A>G, p.Thr2945Ala was rarely found on ExAC (0.0001071) and HGVD (0.0029). Variation of p.Thr2945Ala was not found on iJGVD. These mutations were predicted to be benign to probably damaging with Polyphen2, neutral to deleterious with PROVEAN, and tolerated to damaging with SIFT, and had CADD scores of 15.87 to 44 (Table 1).

**Table 1:** Mutations in the *VPS13D* and the prediction scores

Patient	Mutation	Polyphen2	PROVEAN	SIFT	CADD score	ExAC	HGVD
A‐II‐1	c.1215T>G, p.Ser405Arg	Benign	Neutral	Damaging	15.87	A	A
A‐II‐1	c.9421C>T, p.Arg3141*	NA	NA	NA	44	A	A
B‐II‐1 and B‐II‐2	c.3353C>T p.Thr1118Met	Probably damaging	Deleterious	Damaging	26.5	A	A
C‐II‐2	c.8833A>G p.Thr2945Ala	Benign	Neutral	Tolerated	22	0.0001071	0.0029

Abbreviations: A, absent; NA, not available.

The clinical features of the patients with *VPS13D* mutations are shown in Table 2. Patients B‐II‐1, B‐II‐2, and C‐II‐2 exhibited a pure form of HSP. Patients B‐II‐1 and B‐II‐2 exhibited gait instability in their 40s and Patient C‐II‐2 showed it in her 60s. Patients B‐II‐1, B‐II‐2 and C‐II‐2 showed Babinski signs. These three patients exhibited exaggerated tendon reflexes in their lower limbs. Patient C‐II‐2 exhibited a dexterity movement disorder in both hands in her late 70s. Patient C‐II‐2 and her brothers, C‐II‐3, 4, and 5, showed ichthyosis, which was due to a known mutation of *CYP4F22* (c.728G>A, p.Arg243His) that was also identified on exome analysis of patient C‐II‐1.

**Table 2:** Clinical features of the four patients with *VPS13D* mutations

Patient	A‐II‐1	B‐II‐1	B‐II‐2	C‐II‐1
Age of onset (y.o.)	3	42	40	63
Age of examination (y.o)	42	57	55	71
Phenotype	Complicated HSP	Pure HSP	Pure HSP	Pure HSP
Mental development	Normal	Normal	Normal	Normal
Leg spasticity	+	+	+	+
Exaggerated tendon reflexes	LL	LL	LL	Jaw LL
Babinski sign	+	+	+	+
Ataxia	+	−	−	−
Dystonia	+	−	−	−
Chorea	−	−	−	−
Chorioretinal dystrophy	+	−	−	−

Abbreviations: −, negative; +, positive; LL, lower limb; y.o., years old.

## 4 DISCUSSION

In the present study, we found three patients with an adult onset pure form of HSP and one with a childhood onset complicated form of HSP exhibiting novel *VPS13D* mutations.

The symptoms of one patient (A‐II‐1) are similar to those in earlier reports of *VPS13D*‐related disorders, showing a wide spectrum and variability of severity (Gautheir et al., 2018; Seong et al., 2018). The patient (A‐II‐1) exhibited normal global development, pyramidal signs, cerebellar ataxia, and extrapyramidal signs, which comprise the most frequent phenotype of *VPS13D*‐related disorders (Gautheir et al., 2018; Seong et al., 2018). In addition, the patient showed chorioretinal dystrophy and juvenile bilateral cataracts without known gene variations of retinal dystrophy and cataracts. This might indicate that a *VPS13D* or *ABHD4* mutation causes retinal dystrophy and cataracts. *ABHD4* is one of the ABHD family, and is known as a lysophospholipase selective for N‐acyl phosphatidylethanolamine. *ABHD4* is ubiquitously expressed in multiple tissues, with the highest expression in the brain, small intestine, kidneys, and testes. Mutations of another ABHD family member, *ABHD12* (OMIM *613599), which results in polyneuropathy, hearing loss, ataxia, retinitis pigmentosa, and cataracts (PHARC), could also lead to cataracts. Meanwhile, retinal dystrophy is a major symptom of Cohen syndrome caused by *VPS13B* mutations in the VPS13 family (Kolehmainen et al., 2003), and thus *VPS13D* mutations might cause the retinal symptom. Therefore, Patient A‐II‐1 might be the first case presenting chorioretinal dystrophy caused by *VPS13D*.

Meanwhile, the three other patients revealed a pure form of HSP. To date, HSP has been reported in only one patient (Gautheir et al., 2018). According to earlier reports (Gautheir et al., 2018; Seong et al., 2018), 19 patients exhibited cerebellar ataxia (79%; 15/19), movement disorders (dystonia, chorea, and tremor) (37%; 7/19), cognitive impairment (42%; 8/19), and spastic paraplegia (0.5%; 1/19). Our four patients exhibited spastic paraplegia (100%; 4/4), cerebellar ataxia (25%; 1/4), movement disorders (25%; 1/4), and no cognitive impairment. This suggests that patients with *VPS13D* mutations have a broad clinical spectrum including childhood and adult onset, movement disorders, cerebellar ataxia, spastic ataxia, spastic paraplegia, and hypotonia.

In the present study, we found four novel *VPS13D* mutations (p.Ser405Arg, p.Arg3141Ter, p.Thr1118Met, and p.Thr2945Ala). These mutations other than p.Thr2945Ala were predicted to be disease causing on several in silico analyses. The variation of p.Thr2945Ala was judged to be benign, neutral, and tolerated by Polyphen2, PROVEAN, and SIFT, respectively. Only the CADD score, which was 22, indicated disease causing. To date, 23 mutations in the *VPS13D* have been described (Gautheir et al., 2018; Seong et al., 2018). Earlier studies showed 11 compound heterozygous mutations and one homozygous mutation (Gautheir et al., 2018; Seong et al., 2018).*VPS13D* has 69 coding exons, and 27 reported mutations including ours are widely spread in the *VPS13D* (Gautheir et al., 2018; Seong et al., 2018). Since the frequency of variation of c.8833A>G, p.Thr2945Ala was reported to be extremely low in the control database, there is the possibility that it is a polymorphism. To date, 12 families including ours have been reported. There have only been three families with homozygous missense mutations. Other families had loss‐of‐function mutations. This might indicate that severe changes causing mutations are needed for *VPS13D*‐related disorders. Thus, it is necessary to collect more patients with homozygote *VPS13D* mutations to elucidate the phenotype–genotype correlation.

*VPS13D* mutations were reported to be associated with mitochondrial dysfunction (Seong et al., 2018). Each mutation might lead to mitochondrial dysfunction in a different way. This might lead to phenotype variations such as cognitive impairment, cataracts, chorioretinal dystrophy, ataxia, and spasticity. To clarify any phenotype–genotype correlation, the accumulation of patients with *VPS13D* mutations is required.

## CONFLICT OF INTEREST

None.
