# Supplementary material for: VPS13D‐related disorders presenting as a pure and complicated form of hereditary spastic paraplegia
Source: Mol Genet Genomic Med. 2019 Dec 26;8(3):e1108. doi: 10.1002/mgg3.1108 (PMC7057107; doi:10.1002/mgg3.1108)
Supplement: Supplementary file 1 [file MGG3-8-e1108-s001.docx]

Supplemental information 1.

SNPs and short indels of these genes were screened by whole-exome analysis.

HSPs

*ATL1, SPAST, NIPA1, KIAA0196, ALDH18A1, KIF5A, RTN2, HSPD1, BSCL2, ATSV, REEP1, ZFYVE27, SLC33A1, REEP2, CPT1C, CYP7B1, SPG7, ALDH18A1, SPG11, ZFYVE26, ERLIN2, SPG20, ACP33, B4GALNT1, DDHD1, KIF1A, FA2H, PNPLA6, c9orf12, GJA12, NT5C2, GBA2, AP4B1, KIAA0415, TECPR2, AP4M1, AP4E1, AP4S1, VPS37A, DDHD2, c12orf65, CYP2U1, TFG, KIF1C, USP8, WDR48, ARL6IP1, ERLIN1, AMPD2, ENTPD1, ARSI, PGAP1, FLRT1, RAB3GAP2, MARS, ZFR, IBA57, MAG.*

CMTs

*PMP22, MPZ, LITAF, EGR2, NEFL, FBLN5, KARS, SOX10, GJB3, ARHGEF10, GNB4, HARS, GDAP, MTMR2, SBF2, SBF1, SH3TC2, NDRG1, EGR2, PRX, HK1, FGD4, FIG4, SURF1, CTDP1, ASAH1, PMM2, GALC, ARSA, PHYH, PEX7, ABHD12, DNAJC3, GJB1, MFN2, KIF1B, RAB7, TRPV4, GARS, HSPB1, GDAP1, HSPB8, DNM2, AARS, DYNC1H1, LRSAM1, DHT, DNAJB2, MARS, NAGLU, HARS, VCP, MORC2, NEFH, TFG, DGAT2, MME, DCAF8, SPTLC1, SPTLC2, SPTLC3, IFRD1, BSCL2, TUBB3, LMNA, MED25, HSPB1, GDAP1, LRSAM1, TRIM2, IGHMBP2, MME, SPG11, ATSV, KCC3, SCYL1, TDP1, PLA2G6, GAN, HINT1, SLC25A46, GJB1, PDK3, AIFM1, PRPS1, YARS, INF2, GNB4, ARHGEF10, GDAP, PLEKHG5, COX6A1, DRP2, EGR2, EMILIN1, AIFM1, MTMR2, EGR2, PRX, DHH, HOXD10.*

SCAs

*SPTBN2, ATXN10, TTBK2, PPP2R2B, KCNC3, PRKCG, ITPR1, TBP, KCND3, TMEM240, PDYN, EEF2, FGF14, AFG3L2, ELOVL4, TGM6, ELOVL5, CCDC88C, TRPC3, CACNA1G, MME, GRM1, FAT2, PLD3, PUM1, STUB1, SYNE1, ADCK3(20151021), SYT14, ANO10, APTX, SETX, GRID2, ATM, MRE11, TDP1, DNAJC19, ATCAY, SACS, ERCC8, ERCC6, APTX, COL18A1, WWOX, GOSR2, FXN,*

*C10orf2, PLA2G6, DARS2, SIL1, CSTB, PRICKLE1, SCARB2, KCTD7, ABHD12, MARS2, ACO2, SLC17A5, POLG, KCNJ10, POLR3A, TTPA, MTTP, CYP27A1, SLC6A19, APOB, BCKDHA, BCKDHB, DNAJC19, NPC1, PHYH, ATP7B, KIAA0226, TDP2, SNX14, PIK3R5, EXOSC3, LAMA1, LAMA2, LAMA3, LAMA4, LAMA5, PCNA, TPP1, AARS2, CLCN2, TRAPPC11, GRM1, HPDL*

Retinal dystrophy

*ABCA4, ABHD12, ACBD5, ADAM9, ADAMTS18, ADGRA3, ADGRV1, AHI1, AIPL1, ALMS1, ATF6, BBS1, BBS10, BEST1, BRAT1, C8orf37, CABP4, CACNA1F, CACNA2D4, CASK, CC2D2A, CDH23, CDHR1, CEP290, CERKL, CFAP410, CHM, CLN3, CLRN1, CNGA1, CNGA3, CNGB1, CNGB3, CNNM4, CRB1, CRX, CSNK2A1, CTNNA1, CYP4V2, DDHD1, DRAM2, EFEMP1, EFTUD2, EYS, FAM161A, FLVCR1, GJA9, GRID2, GRK1, GUCA1A, GUCA1B, GUCY2D, HGSNAT, HOOK2, IFT140, IMPG2, INPP5E, IQCB1, ITM2B, KCNJ13, KCNV2, KIF11, LCA5, LRAT, LRP2, LRP5, LTBP2, MAK, MERTK, MFRP, MFSD8, MIR204, MVK, MYO7A, NPHP1, NPHP4, NR2E3, NRL, NYX, OAT, OCA2, orf15, OTX2, P3H2, PAX2, PCARE, PCDH15, PCYT1A, PDE6A, PDE6B, PDE6C, PHF3, PIPNM3, PROM1, PRPF31, PRPF6, PRPF8, PRPH2, PRPS1, RAX2, RBP3, RBP4, RCBTB1, RD3, RDH12, RGR, RHO, RLBP1, RP1, RP1L1, RP2, RPE65, RPGR, RPGRIP1, RS1, SLC19A2, SNRNP200, SPATA7, SRD5A3, SVOPL, TOPORS, TRPM1, ttc8r, TTLL5, TUB, TULP1, USH1C, USH1G, USH2A, VPS13B, WDPCP, ZNF674*

Cataracts

*ABCA3, ABHD12, ACKR1, ADAM9, ADAMTS18, ADD3, AGK, AKR1E2, ALDH18A1, APP, BCOR, BEST1, BFSP1, BFSP2, BMP4, BRD4, CDK5RAP2, CHD7, CHMP4B, CLPB, COL4A1, COL4A2, CRYAA, CRYAB, CRYBA1, CRYBA2, CRYBA4, CRYBB1, CRYBB2, CRYBB3, CRYGA, CRYGB, CRYGC, CRYGD, CRYGS, CTDP1, CYP1B1, CYP27A1, CYP51A1, DNA2, DNM2, DNMBP, DYNC1H1, EFNA5, EIF2B2, EPHA2, ERCC2, EYA1, EZR, FAM126A, FAR1, FBN1, FOXE3, FTL, FYCO1, GALE, GALK1, GALT, GCNT2, gcnt2c, GDF3, GEMIN4, GFER, GJA3, GJA8, GLS, GNPAT, GSR, GSTM1, GSTT1, HSF4, hsf4b, IARS2, IDO1, INPP5K, JAM3, KCNA4, KCNJ13, LEMD2, LIM2, LONP1, LSS, MAF, MED13, MFSD6L, MIP, MIR184, MVK, MYH9, MYOC, NACC1, NECAP2, NECTIN3, NHS, OCRL, OGG1, OPA1, OPA3, PARK7, PAX6, PEX11B, PIGY, pitx2c, PITX3, POLG, PRX, PXDN, RGS6, RIC1, RIMS1, RNLS, RRAGA, RRM2B, RYR1, SC5D, SIL1, SIPA1L3, SIX5, SLC16A12, SLC33A1, SLC40A1, SLURP1, STX3, TAF1A, TAPT1, TDRD7, TFR2, TMCO3, TMEM114, TMEM70, TRAPPC11, TRNT1, TRPM3, trpm3tv9, TUBA1A, TUBB, UCHL1, UNC45B, VIM, VSX2, WDR36, WDR87, WFS1, WRN, XYLT2, ZNF350*
