# Supplementary material for: VPS13D‐related disorders presenting as a pure and complicated form of hereditary spastic paraplegia
Source: Mol Genet Genomic Med. 2019 Dec 26;8(3):e1108. doi: 10.1002/mgg3.1108 (PMC7057107; doi:10.1002/mgg3.1108)
Supplement: Supplementary file 2 [file MGG3-8-e1108-s002.docx]

Supplemental information 2.

Repeat expansions were checked in these genes.

SCAs

*SCA1, SCA2, MJD/SCA3, SCA6, SCA7, SCA8, SCA12, SCA17, DRPLA*
